# Association of the triglyceride-glucose index and vascular target organ damage in a Beijing community-based population

**DOI:** 10.3389/fcvm.2022.948402

**Published:** 2022-07-28

**Authors:** Wenjun Ji, Lan Gao, Pengfei Sun, Jia Jia, Jianping Li, Xingang Wang, Fangfang Fan, Yan Zhang

**Affiliations:** ^1^Department of Cardiology, Peking University First Hospital, Beijing, China; ^2^Institute of Cardiovascular Disease, Peking University First Hospital, Beijing, China; ^3^Echocardiography Core Lab, Institute of Cardiovascular Disease at Peking University First Hospital, Beijing, China

**Keywords:** the triglyceride glucose index, vascular target organ damage, carotid-femoral pulse wave velocity, brachial-ankle pulse wave velocity, urine albumin-to-creatinine ratio

## Abstract

**Objective:**

We aimed to explore the association between the triglyceride-glucose (TyG) index, a marker of insulin resistance (IR), and vascular target organ damage (TOD) in a Beijing community-based population, China.

**Methods:**

A total of 6,015 participants from an atherosclerosis cohort survey performed in the Shijingshan District in Beijing, China were included in our analysis. Vascular TOD, such as carotid-femoral pulse wave velocity (cfPWV), brachial-ankle pulse wave velocity (baPWV), and the urine albumin-to-creatinine ratio (UACR) were all evaluated.

**Results:**

The overall mean age of all the participants was 62.35 years, 3,951 (65.69%) were female, and mean TyG index was 8.81. In univariable regression analyzes, an increased TyG index was associated with higher cfPWV, baPWV, lnUACR, and higher risk of cfPWV ≥ 10 m/s, baPWV ≥ 1,800 cm/s, and UACR ≥ 30 mg/g, respectively. Multivariable regression analyzes showed subjects with the TyG index in top tertile had a significant increase in cfPWV (β = 0.29 m/s; 95% confidence interval [95% *CI*] 0.19–0.40; *p*_fortrend_ < 0.001), baPWV (β = 69.28 cm/s; 95% *CI* 50.97–87.59; *p*_fortrend_ < 0.001), lnUACR (β = 0.23; 95% *CI* 0.13–0.34; *p*_fortrend_ < 0.001), and had a higher risk of cfPWV ≥ 10 m/s (odds ratio [*OR*] = 1.47; 95% *CI* 1.17–1.85; *p*_fortrend_ < 0.001), baPWV ≥ 1,800 cm/s (*OR* = 1.79; 95% *CI* 1.48–2.17; *p*_fortrend_ < 0.001), and UACR ≥ 30 mg/g (*OR* = 1.71; 95% *CI* 1.30–2.24; *p*_fortrend_ < 0.001) after fully adjusting for age, sex, body mass index (BMI), systolic blood pressure (SBP), diastolic blood pressure (DBP), high-density lipoprotein cholesterol (HDL-C), low-density lipoprotein cholesterol (LDL-C), estimated glomerular filtration rate (eGFR), self-reported coronary heart disease (CHD) and stroke, antihypertensive drugs, hypoglycemic drugs, and lipid-lowering drugs. Consistent conclusions were obtained in the subgroups without hypoglycemic and lipid-lowering medications or aged younger than 65 years old.

**Conclusions:**

The TyG index was positively associated with artery stiffness and nephric microvascular damage in a Beijing community-based population in China. This result provides evidence that the TyG index may serve as a simple and effective indicator to reflect vascular TOD.

## Introduction

Defined as a decrease in tissue response to insulin stimulation, insulin resistance (IR) is associated with an increased risk of metabolic abnormalities, such as hyperglycemia, dyslipidemia, and hypertension, and is closely associated with cardiovascular disease (CVD) (1, 2). The gold standard “Euglycemic clamp test” is cumbersome, expensive, and non-feasible in routine clinical setups for evaluating IR in clinical practice (3). Recently, the triglyceride-glucose (TyG) index, calculated as Ln (fasting triglycerides (mg/dl) × fasting plasma glucose (mg/dl)/2), became an attractive option due to the high availability and cheap biochemical markers required for their calculations, and the diagnostic accuracy of the TyG index in identifying IR has been tested in several studies (4, 5).

Vascular injury related to IR develops progressively in asymptomatic subjects during a period of time, and the long phase of IR and presence of subclinical vascular disease increase cardiovascular risk (2, 6–8). The vascular injury involves functional and structural damage to the arterial wall that includes impaired vasodilation in response to chemical mediators, reduced distensibility of the arterial wall, vascular calcification, and increased thickness of the arterial wall (8, 9). The previous studies have shown that IR was associated with arterial stiffness (10–13), atherosclerosis (14, 15), and microcirculation lesions (16–18). Wen et al. found the TyG index is independently associated with arterial stiffness and 10-year CVD risk (19). Lv et al. found that the TyG index is a potential predictor for diabetic kidney disease (DKD) in type 2 diabetes mellitus (T2DM) patients (18).

At present, there are few systematic studies on TyG index and the various vascular damages of IR. This study explored the association of vascular target organ damage (TOD) with TyG index in a Beijing community-based population.

## Materials and methods

### Study population

Participants came from an atherosclerosis cohort survey conducted in the Gucheng and Pingguoyuan communities of Shijingshan District in Beijing, China from December 2011 to April 2012. Detailed research procedures have been described previously (20). Subjects (*n* = 6,568) who participated in the 7th year of on-site follow-up from September 2018 to December 2018 were enrolled in our analyses. This study was restricted to a subset of participants with brachial-ankle pulse wave velocity (baPWV), carotid-femoral pulse wave velocity (cfPWV), and urine albumin-to-creatinine ratio (UACR) data available at baseline (*n* = 6,329). Participants with any self-reported history of peripheral artery disease (PAD) or ankle-brachial index (ABI) < 0.90 were excluded (*n* = 314). Finally, a total of 6,015 eligible participants were ultimately included. This study was approved by the ethics committee of Peking University First Hospital, and it conforms to the provisions of the Declaration of Helsinki. All participants signed informed consent.

### Clinical data collection

#### Baseline data collection

The standard questionnaire survey data such as sociodemographic characteristics, lifestyle, detailed medical information, and etc., were collected by uniformly trained investigators in the community health service center using a face-to-face inquiry survey method. Current smoking was defined as smoking one or more cigarettes per day for at least 6 months. Current drinking was defined as drinking once per week for at least 6 months. Hypertension was defined as any systolic blood pressure (SBP) ≥ 140 mmHg or diastolic blood pressure (DBP) ≥ 90 mmHg or self-reported history of hypertension or use of antihypertensive medication. Diabetes mellitus was defined as any fasting plasma glucose (FBG) ≥ 7.0 mmol/L, or 2-h oral glucose tolerance test (OGTT) ≥ 11.1 mmol/L or self-reported history of diabetes, or use of hypoglycemic medication. Dyslipidemia was defined as any triglyceride (TG) ≥ 1.7 mmol/L (150 mg/dl), or total cholesterol (TC) ≥ 5.18 mmol/L (200 mg/dl), or low-density lipoprotein cholesterol (LDL-C) ≥ 3.37 mmol/L (130 mg/dl), or high-density lipoprotein cholesterol (HDL-C) < 1.04 mmol/L (40 mg/dl), or self-reported history of dyslipidemia, or use of lipid-lowering medication. The history of CVD was defined as any self-reported history of coronary heart disease, stroke, or transient ischemic attack.

Physical examination data, such as height and weight, were collected by uniformly trained surveyors according to the standard operating procedures. The body mass index (BMI) was calculated by the following formula: BMI = weight (kg)/height (2) (m^2^). The SBP and DBP used were the mean value of these three successful readings measured by Omron HEM-7130 electronic sphygmomanometer by a standard method (20).

#### Laboratory examination

Venous blood samples were obtained in subjects after an overnight fast. Biological markers, such as FPG, the standard 75 g OGTT, TG, TC, low-density lipoprotein cholesterol (LDL-C), high-density lipoprotein cholesterol (HDL-C), and serum creatinine (Scr), were measured by enzymatic method (HITACHI 7100, HITACHI, Japan). The estimated glomerular filtration rate (eGFR) was calculated based on the CKD-EPI equation (21). Urine samples required subjects to take 15 ml of morning urine on the day of the survey. Urine microalbumin was assessed by the rate scattering turbidimetry method (Immang 800, Beckman, USA), and urine creatinine was analyzed by the picric acid method (AU5800, Beckman, USA), and urinary microalbumin divided by urinary creatinine was defined as UACR.

#### Pulse-wave velocity

In this study, baPWV was measured by the BP-203RPEIII (Omron, Omron Healthcare, Japan) device. After the subject rested in the supine position for at least 5 min, cuffs were wrapped on the upper arms and ankles, and the pulse waves of brachial and posterior tibial arteries at the cuffs were recorded by the device. The distance between the upper arm and ankle is calculated using a liner regression of body height and baPWV on both sides was obtained using the distance divided by the time difference *via* the device automatically. The higher of the bilateral baPWV was selected for the subsequent analysis. The details of the oscillometric method have been described and validated previously (22).

The CfPWV was measured using PulsePen (DiaTecne, Italy) systems in accordance with standard operating procedures after resting in the supine position for at least 5 min. The pulse waveforms of the strongest beating points of each participant's right carotid and femoral were collected. The distances from carotid to femoral, carotid to sternal angle, and sternal angle to femoral were measured simultaneously with a ruler and the direct carotid–femoral distance between the two recording sites was calculated as (common carotid artery – common femoral artery × 0.8) tape measure distance. The pass time was calculated by the “foot-to-foot” method that was taken into the device for automatic calculation of cfPWV. The CfPWV was performed twice, and the average value was used. If the difference between the two measurements exceeds 0.5 m/s, a third measurement was undertaken and the average of the three measurements was adopted.

#### Definition of TOD

Carotid-femoral pulse wave velocity, mainly reflects the elasticity of aorta, and baPWV, mainly reflects the elasticity of large and middle arteries, act as two most frequently applied indicators to evaluate arteriosclerosis (23). According to the expert consensus document, we defined 10 m/s (24) and 1,800 cm/s (25) as the cut-off value for cfPWV and baPWV, respectively. The UACR is a commonly used clinical indicator to reflect renal microvascular damage and microalbuminuria was defined as UACR ≥ 30 mg/g.

### Statistical analyses

Descriptive statistics were expressed as mean ± standard deviation (*SD*) or median (interquartile range) for continuous variables as appropriate and frequencies (percentage) for categorical variables. The baseline characteristics of the different groups by TyG index tertiles were compared using the ANOVA or Kruskal–Wallis *h*-test when appropriate for the continuous variables, and the chi-square test or Fisher's exact test (if the theoretical number < 10) for the categorical variables. The independent associations of the TyG index with TOD were evaluated using univariable and multivariable regression models with adjustment for major covariables in model 1 and model 2. Model 1 was adjusted for age and sex. Model 2 was further adjusted for other clinical variables, such as BMI, SBP, DBP, HDL-C, LDL-C, antihypertensive drugs, hypoglycemic drugs, lipid-lowering drugs, self-reported coronary heart disease (CHD), and stroke. In addition, the associations between TyG index and TOD were also evaluated by sensitivity analyses in subgroups without using hypoglycemic and lipid-lowering medications or those aged younger than 65 years old. All data analyses were using Empower (R) (www.empowerstats.com, X&Y solutions, inc.BostonMA) and R (Version: 3.4.3; http://www.R-project.org). A 2-tailed *p* < 0.05 was considered to be statistically significant.

## Results

### Baseline characteristics of all participants according to the TyG index tertiles

A total of 6,015 participants were included in the current analysis. Table 1 shows the clinical and laboratory characteristics of the study population by the TyG index tertiles, with 2001, 2009, and 2005 participants in each group, respectively. The mean values ± *SDs* of the TyG index in the three groups were 8.19 ± 0.25, 8.76 ± 0.14, and 9.48 ± 0.43, respectively. There were statistically differences in age, gender, BMI, SBP, DBP, Glu, TC, TG, HDL-C, LDL-C, smoking habit, prevalence of CHD, hypertension, diabetes, and dyslipidemia, using of antihypertensive, hypoglycemic, and lipid-lowering drugs, cfPWV, cfPWV ≥ 10 m/s, baPWV, baPWV ≥ 1,800 cm/s, UACR, and UACR ≥ 30 mg/g among the three groups, while there were no differences in eGFR, drinking habit, and stroke prevalence.

**Table 1:** Baseline characteristics of the study population according to the tertiles of the triglyceride glucose (TyG) index.

	**Total**	**Tertile 1 (< 8.52)**	**Tertile 2 (≥ 8.52–< 9.01)**	**Tertile 3 (≥ 9.01)**	***P value***
N	6,015	2,001	2,009	2,005	
Age (years)	62.35 ± 7.60	62.37 ± 8.18	62.63 ± 7.43	62.05 ± 7.14	0.031
Female, n (%)	3,951 (65.69%)	1,294 (64.67%)	1,370 (68.19%)	1,287 (64.19%)	0.014
BMI (kg/m^2^)	25.23 ± 3.31	24.19 ± 3.27	25.37 ± 3.28	26.13 ± 3.08	<0.001
SBP (mmHg)	133.02 ±16.62	129.91 ± 16.57	132.87 ± 16.22	136.28 ± 16.45	<0.001
DBP (mmHg)	79.02 ± 9.54	77.42 ± 9.57	78.73 ± 9.36	80.91 ± 9.36	<0.001
Fasting blood glucose(mmol/L)	6.16 ± 1.87	5.43 ± 0.75	5.88 ± 1.20	7.18 ± 2.62	<0.001
TC (mmol/L)	5.33 ± 1.03	5.08 ± 0.95	5.33 ± 1.03	5.59 ± 1.05	<0.001
TG (mmol/L)	1.37 (0.98–1.94)	0.87 (0.72–1.02)	1.40 (1.23–1.60)	2.28 (1.90–2.87)	<0.001
HDL-C (mmol/L)	1.50 ± 0.35	1.68 ± 0.39	1.49 ± 0.30	1.32 ± 0.26	<0.001
LDL-C (mmol/L)	3.42 ± 0.97	3.23 ± 0.87	3.53 ± 0.99	3.51 ± 1.02	<0.001
EGFR (mL/min per 1.73 m^2^)	93.29 ± 11.47	93.33 ± 11.50	93.27 ± 10.95	93.26 ± 11.95	0.978
TyG index	8.81 ± 0.61	8.19 ± 0.25	8.76 ± 0.14	9.48 ± 0.43	<0.001
Current smoking, n (%)	839 (14.12%)	237 (11.98%)	244 (12.28%)	358 (18.09%)	<0.001
Current drinking, n (%)	629 (10.55%)	193 (9.71%)	200 (10.01%)	236 (11.94%)	0.129
Self-reported disease, n (%)					
CHD	718 (11.96%)	195 (9.76%)	251 (12.50%)	272 (13.62%)	<0.001
Stroke	264 (4.40%)	81 (4.05%)	86 (4.28%)	97 (4.86%)	0.440
Disease, n (%)					
Hypertension	3,286 (54.63%)	910 (45.48%)	1,079 (53.71%)	1,297 (64.69%)	<0.001
Diabetes	1,741 (28.95%)	269 (13.44%)	499 (24.84%)	973 (48.55%)	<0.001
Dyslipidemia	4,871 (80.98%)	1,293 (64.62%)	1,639 (81.58%)	1,939 (96.71%)	<0.001
Treatment, n (%)					
Antihypertensive drugs	2,164 (36.08%)	577 (28.92%)	724 (36.06%)	863 (43.26%)	<0.001
Hypoglycemic drugs	976 (16.26%)	166 (8.32%)	272 (13.54%)	538 (26.95%)	<0.001
Lipid-lowering drugs	1,199 (20.01%)	287 (14.40%)	419 (20.89%)	493 (24.72%)	<0.001
Target organ damage					
CfPWV (m/s)	8.56 ± 1.84	8.29 ± 1.72	8.53 ± 1.86	8.86 ± 1.89	<0.001
CfPWV ≥ 10 m/s, n (%)	1,028 (17.09%)	275 (13.74%)	339 (16.87%)	414 (20.65%)	<0.001
BaPWV (cm/s)	1,680.16 ± 335.80	1,617.64 ± 321.55	1,677.87 ± 328.78	1,744.86 ± 344.67	<0.001
BaPWV ≥ 1,800 cm/s, n (%)	1,826 (30.36%)	477 (23.84%)	601 (29.92%)	748 (37.31%)	<0.001
UACR (mg/g)	3.16 (1.58–8.48)	2.76 (1.41–6.36)	3.02 (1.57–7.27)	3.98 (1.83–12.44)	<0.001
LnUACR	1.36 ± 1.47	1.15 ± 1.35	1.29 ± 1.40	1.63 ± 1.59	<0.001
UACR ≥ 30 mg/g, n (%)	529 (8.79%)	123 (6.15%)	142 (7.07%)	264 (13.17%)	<0.001

Data are shown as mean ± standard deviation (SD) or median (IQR) for continuous variables and number (percentage) for dichotomous variables.

BMI, body mass index; SBP, systolic blood pressure; DBP, diastolic blood pressure; TC, total cholesterol; TG, triglycerides; HDL-C, high-density lipoprotein cholesterol; LDL-C, low-density lipoprotein cholesterol; eGFR, estimated glomerular filtration rate; TyG, triglyceride glucose; CHD, coronary heart disease; cfPWV, carotid to femoral aortic pulse wave velocity; baPWV, brachial to ankle pulse wave velocity; UACR, urine albumin to creatinine ratio.

### The associations between TOD and TyG index

**Figure 1:**
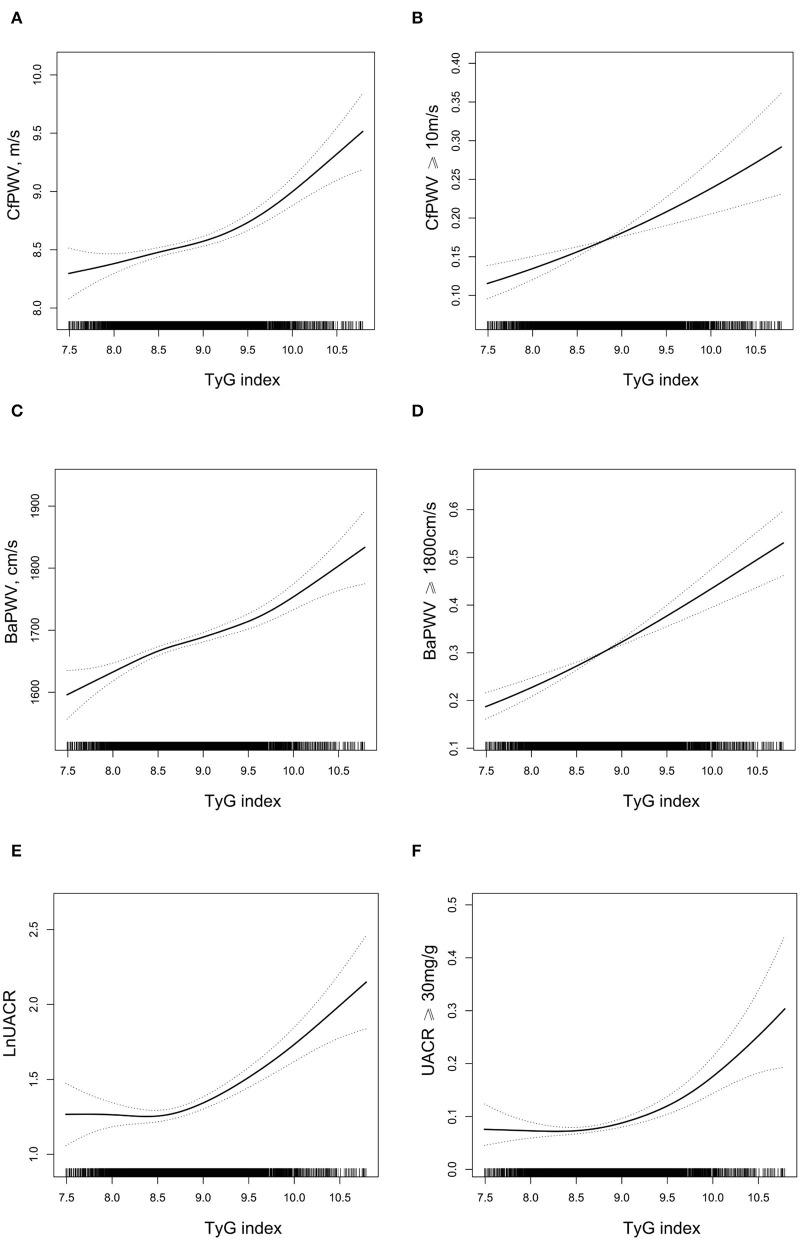
The relationship between triglyceride glucose (TyG) index and target organ damage (TOD)^*^. **(A)** carotid-femoral pulse wave velocity (cfPWV); **(B)** cfPWV ≥ 10 m/s; **(C)** brachial-ankle pulse wave velocity (BaPWV); **(D)** BaPWV ≥ 1,800 cm/s; **(E)** LnUACR; **(F)** urine albumin-to-creatinine ratio (UACR) ≥ 30 mg/g. ^*^Adjusted for age, sex, smoking, and drinking habit, body mass index (BMI), systolic blood pressure (SBP), diastolic blood pressure (DBP), high-density lipoprotein cholesterol (HDL-C), low-density lipoprotein cholesterol (LDL-C), estimated glomerular filtration rate (eGFR), self-reported coronary heart disease (CHD) and stroke, antihypertensive drugs, hypoglycemic drugs, lipid-lowering drugs. A 2-tailed 0.5% of TyG index was removed.

As shown in Figure 1, cfPWV, baPWV, lnUACR, the odds of cfPWV ≥ 10 m/s, baPWV ≥ 1,800 cm/s, and UACR ≥ 30 mg/g all increased with the increase of TyG index after adjustment for traditional cardiovascular risk factors.

In Table 2, for per 1 unit increment in TyG index, cfPWV, baPWV, and lnUACR increased by 0.29 m/s (95% *CI* 0.21, 0.36, *p* < 0.001), 58.36 cm/s (95% *CI* 45.83, 70.89, *p* < 0.001), and 0.23 cm/s (95% *CI* 0.16, 0.30, *p* < 0.001), respectively; the odds of cfPWV ≥ 10 m/s, baPWV ≥ 1,800 cm/s, and UACR ≥ 30 mg/g were elevated by 43% (odds ratio [*OR*] = 1.43; 95% *CI* 1.23–1.67; *p* < 0.001), 61% (*OR* = 1.61; 95% *CI* 1.42–1.84; *p* < 0.001), and 77% (*OR* = 1.77; 95% *CI* 1.49–2.10; *p* < 0.001), respectively, after fully adjusting for age, sex, BMI, SBP, DBP, HDL-C, LDL-C, eGFR, antihypertensive drugs, hypoglycemic drugs, lipid-lowering drugs, self-reported CHD, and stroke (adjusted model 2).

**Table 2:** Univariable or multivariable regressions for target organ damage (TOD) according to the TyG index and the tertiles of TyG index.

	**Non-adjusted**	**Adjust model 1**	**Adjust model 2**
	**ß/OR (95%CI)**	**ß/OR (95%CI)**	**ß/OR (95%CI)**
CfPWV, m/s			
Per 1 unit increase	0.46 (0.39, 0.54) ^**^	0.50 (0.43, 0.57) ^**^	0.29 (0.21, 0.36) ^**^
Tertiles of TyG			
Tertile 1 (<8.52)	0	0	0
Tertile 2 (≥ 8.52– <9.01)	0.24 (0.13, 0.35) ^**^	0.23 (0.13, 0.33) ^**^	0.11 (0.01, 0.21) ^*^
Tertile 3 (≥ 9.01)	0.57 (0.46, 0.68) ^**^	0.60 (0.50, 0.70) ^**^	0.29 (0.19, 0.40) ^**^
*P* for trend	<0.001	<0.001	<0.001
CfPWV ≥ 10 m/s			
Per 1 unit increase	1.48 (1.33, 1.65) ^**^	1.82 (1.62, 2.06) ^**^	1.43 (1.23, 1.67) ^**^
Tertiles of TyG			
Tertile 1 (<8.52)	1.0	1.0	1.0
Tertile 2 (≥ 8.52– <9.01)	1.27 (1.07, 1.51) ^*^	1.43 (1.18, 1.73) ^*^	1.23 (0.99, 1.52)
Tertile 3 (≥ 9.01)	1.63 (1.38, 1.93) ^**^	2.10 (1.74, 2.53) ^**^	1.47 (1.17, 1.85) ^**^
*P* for trend	<0.001	<0.001	<0.001
BaPWV, cm/s			
Per 1 unit increase	95.76 (81.96, 109.55) ^**^	104.51 (92.48, 116.55) ^**^	58.36 (45.83, 70.89) ^**^
Tertiles of TyG			
Tertile 1 (<8.52)	0	0	0
Tertile 2 (≥ 8.52– <9.01)	60.22 (39.68, 80.76) ^**^	55.36 (37.39, 73.33) ^**^	29.65 (13.04, 46.25) ^**^
Tertile 3 (≥ 9.01)	127.22 (106.67, 147.77) ^**^	133.84 (115.86, 151.81) ^**^	69.28 (50.97, 87.59) ^**^
*P* for trend	<0.001	<0.001	<0.001
BaPWV ≥ 1,800 cm/s			
Per 1 unit increase	1.60 (1.46, 1.75) ^**^	1.93 (1.74, 2.13) ^**^	1.61 (1.42, 1.84) ^**^
Tertiles of TyG			
Tertile 1 (< 8.52)	1.0	1.0	1.0
Tertile 2 (≥ 8.52– < 9.01)	1.36 (1.19, 1.57) ^**^	1.46 (1.25, 1.70) ^**^	1.32 (1.11, 1.58) ^*^
Tertile 3 (≥ 9.01)	1.90 (1.66, 2.18) ^**^	2.34 (2.01, 2.73) ^**^	1.79 (1.48, 2.17) ^**^
*P* for trend	<0.001	<0.001	<0.001
LnUACR			
Per 1 unit increase	0.38 (0.32, 0.44) ^**^	0.38 (0.32, 0.44) ^**^	0.23 (0.16, 0.30) ^**^
Tertiles of TyG			
Tertile 1 (<8.52)	0	0	0
Tertile 2 (≥ 8.52– <9.01)	0.14 (0.05, 0.23) ^*^	0.13 (0.04, 0.22) ^*^	0.03 (-0.07, 0.12)
Tertile 3 (≥ 9.01)	0.48 (0.39, 0.57) ^**^	0.48 (0.39, 0.57) ^**^	0.23 (0.13, 0.34) ^**^
*P* for trend	<0.001	<0.001	<0.001
UACR ≥ 30 mg/g			
Per 1 unit increase	2.00 (1.75, 2.29) ^**^	2.14 (1.86, 2.46) ^**^	1.77 (1.49, 2.10) ^**^
Tertiles of TyG			
Tertile 1 (<8.52)	1.0	1.0	1.0
Tertile 2 (≥ 8.52– <9.01)	1.16 (0.90, 1.49)	1.17 (0.91, 1.51)	1.02 (0.78, 1.34)
Tertile 3 (≥ 9.01)	2.32 (1.85, 2.90) ^**^	2.45 (1.95, 3.07) ^**^	1.71 (1.30, 2.24) ^**^
*P* for trend	<0.001	<0.001	<0.001

Model 1: adjusted for age, sex.

Model 2: adjusted for age, sex, smoking and drinking habits, BMI, SBP, DBP, HDL-C, LDL-C, eGFR, self-reported CHD and stroke, antihypertensive drugs, hypoglycemic drugs, lipid-lowering drugs.

TyG, triglyceride glucose; OR, odds ratio; CI, confidence interval.

* p < 0.05;

** p < 0.001; other abbreviations as in Table 1.

The associations between the TyG index and TOD were similar as above when further explored by categorizing the TyG index levels into tertiles and using the bottom tertile as a reference (Table 2). In the model 2, the adjusted coefficients for cfPWV, baPWV, and lnUACR of participants in the top tertile were 0.29 m/s (95% *CI* 0.19, 0.40), 69.28 cm/s (95% *CI* 50.97, 87.59), and 0.23 (95% *CI* 0.13, 0.34), respectively. The *p*_fortrend_ in all models was significant (<0.001). Moreover, we found that participants with TyG index in the top tertile had increased risk of cfPWV ≥ 10 m/s (*OR* = 1.47; 95% *CI* 1.17–1.85; *p*_*fortrend*_ < 0.001), baPWV ≥ 1,800 cm/s (*OR* = 1.79; 95% *CI* 1.48–2.17; *p*_*fortrend*_ < 0.001), and UACR ≥ 30 mg/g (*OR* = 1.71; 95% *CI* 1.30–2.24; *p*_*fortrend*_ < 0.001) compared with those with TyG index in the bottom tertile.

### Sensitivity analysis

We further performed analyses in subgroups not taking both anti-hyperglycemic and lipid-lowering drugs (subgroup 1) or those aged younger than 65 years (subgroup 2), and the results were similar as above (Table 3). Although *p*_fortrend_ was marginally significant for UACR ≥ 30 mg/g, the *ORs* for participants in the middle and top tertile of TyG index in the subgroup 2 were 1.07 (95% *CI* 0.75, 1.52) and 1.40 (95% *CI* 0.97, 2.02), respectively, with an increasing trend, and similar profile for cfPWV ≥ 10 m/s was obtained in subgroup 2.

**Table 3:** The association between TyG index and TOD in subgroups.

	**Non-adjusted**	**Adjust model 1**	**Adjust model 2**
	β**/OR(95%CI)**	β**/OR(95%CI)**	β**/OR(95%CI)**
**No-hypoglycemic and lipid-lowering drugs (*n* = 4,159)**			
CfPWV, m/s			
Per 1 unit increase	0.34 (0.25, 0.43) ^**^	0.39 (0.31, 0.47) ^**^	0.23 (0.14, 0.31) ^**^
Tertiles of TyG index			
Tertile 1 (<8.45)	0	0	0
Tertile 2 (≥ 8.45– <8.92)	0.19 (0.06, 0.32) ^*^	0.18 (0.07, 0.29) ^*^	0.09 (-0.02, 0.20)
Tertile 3 (≥ 8.92)	0.38 (0.25, 0.51) ^**^	0.45 (0.34, 0.56) ^**^	0.23 (0.11, 0.34) ^**^
*P* for trend	<0.001	<0.001	<0.001
CfPWV ≥ 10 m/s			
Per 1 unit increase	1.26 (1.09, 1.46) ^*^	1.59 (1.35, 1.88) ^**^	1.38 (1.13, 1.70) ^*^
Tertiles of TyG index			
Tertile 1 (<8.45)	1.0	1.0	1.0
Tertile 2 (≥ 8.45– <8.92)	1.18 (0.95, 1.47)	1.29 (1.01, 1.65) ^*^	1.20 (0.91, 1.57)
Tertile 3 (≥ 8.92)	1.35 (1.09, 1.67) ^*^	1.83 (1.44, 2.32) ^**^	1.55 (1.17, 2.07) ^*^
*P* for trend	0.006	<0.001	0.002
BaPWV, cm/s			
Per 1 unit increase	82.05 (64.77, 99.33) ^**^	94.53 (79.52, 109.54) ^**^	43.92 (29.00, 58.84) ^**^
Tertiles of TyG index			
Tertile 1 (<8.45)	0	0	0
Tertile 2 (≥ 8.45– <8.92)	61.58 (37.21, 85.94) ^**^	57.69 (36.52, 78.86) ^**^	33.36 (14.31, 52.40) ^**^
Tertile 3 (≥ 8.92)	104.59 (80.24, 128.94) ^**^	119.17 (98.01, 140.33) ^**^	56.62 (35.90, 77.34) ^**^
*P* for trend	<0.001	<0.001	<0.001
BaPWV ≥ 1,800 cm/s			
Per 1 unit increase	1.48 (1.31, 1.66) ^**^	1.84 (1.61, 2.10) ^**^	1.49 (1.26, 1.77) ^**^
Tertiles of TyG index			
Tertile 1 (<8.45)	1.0	1.0	1.0
Tertile 2 (≥ 8.45– <8.92)	1.37 (1.15, 1.63) ^**^	1.47 (1.21, 1.79) ^**^	1.38 (1.10, 1.73) ^*^
Tertile 3 (≥ 8.92)	1.70 (1.43, 2.01) ^**^	2.22 (1.83, 2.68) ^**^	1.73 (1.36, 2.20) ^**^
*P* for trend	<0.001	<0.001	<0.001
LnUACR			
Per 1 unit increase	0.26 (0.19, 0.33) ^**^	0.27 (0.20, 0.34) ^**^	0.14 (0.06, 0.23) ^**^
Tertiles of TyG index			
Tertile 1 (<8.45)	0	0	0
Tertile 2 (≥ 8.45– <8.92)	0.12 (0.02, 0.23) ^*^	0.12 (0.02, 0.22) ^*^	0.04 (-0.07, 0.14)
Tertile 3 (≥ 8.92)	0.32 (0.22, 0.42) ^**^	0.33 (0.23, 0.43) ^**^	0.15 (0.03, 0.27) ^*^
*P* for trend	<0.001	<0.001	0.011
UACR ≥ 30 mg/g			
Per 1 unit increase	1.56 (1.29, 1.89) ^**^	1.69 (1.38, 2.06) ^**^	1.45 (1.15, 1.84) ^*^
Tertiles of TyG index			
Tertile 1 (<8.45)	1.0	1.0	1.0
Tertile 2 (≥ 8.45– <8.92)	1.15 (0.84, 1.58)	1.16 (0.84, 1.59)	1.01 (0.72, 1.41)
Tertile 3 (≥ 8.92)	1.70 (1.27, 2.28) ^**^	1.84 (1.37, 2.48) ^**^	1.41 (1.00, 1.98)
*P* for trend	<0.001	<0.001	0.037
**Age <65 years (*n* = 4,023)**			
CfPWV, m/s			
Per 1 unit increase	0.50 (0.43, 0.58) ^**^	0.46 (0.39, 0.53) ^**^	0.25 (0.18, 0.33) ^**^
Tertiles of TyG index			
Tertile 1 (<8.53)	0	0	0
Tertile 2 (≥ 8.53– <9.03)	0.21 (0.10, 0.31) ^**^	0.16 (0.05, 0.26) ^*^	0.06 (-0.04, 0.16)
Tertile 3 (≥ 9.03)	0.63 (0.52, 0.74) ^**^	0.55 (0.45, 0.66) ^**^	0.26 (0.15, 0.37) ^**^
*P* for trend	<0.001	<0.001	<0.001
CfPWV ≥ 10 m/s			
Per 1 unit increase	1.74 (1.48, 2.05) ^**^	1.76 (1.48, 2.09) ^**^	1.29 (1.04, 1.60) ^*^
Tertiles of TyG index			
Tertile 1 (<8.53)	1.0	1.0	1.0
Tertile 2 (≥ 8.53– <9.03)	1.36 (1.01, 1.81) ^*^	1.31 (0.97, 1.75)	1.15 (0.83, 1.60)
Tertile 3 (≥ 9.03)	2.06 (1.57, 2.71) ^**^	1.97 (1.49, 2.59) ^**^	1.31 (0.93, 1.85)
*P* for trend	<0.001	<0.001	0.114
BaPWV, cm/s, ß (95% CI)			
Per 1 unit increase	104.19 (90.35, 118.02) ^**^	99.33 (86.04, 112.63) ^**^	48.95 (35.55, 62.36) ^**^
Tertiles of TyG index			
Tertile 1 (<8.53)	0	0	0
Tertile 2 (≥ 8.53– <9.03)	60.66 (39.72, 81.60) ^**^	49.79 (29.68, 69.90) ^**^	24.54 (6.49, 42.58) ^*^
Tertile 3 (≥ 9.03)	144.31 (123.37, 165.25) ^**^	132.78 (112.63, 152.92) ^**^	64.63 (44.73, 84.52) ^**^
*P* for trend	<0.001	<0.001	<0.001
BaPWV ≥ 1,800 cm/s			
Per 1 unit increase	1.94 (1.72, 2.20) ^**^	2.00 (1.76, 2.28) ^**^	1.62 (1.37, 1.91) ^**^
Tertiles of TyG index			
Tertile 1 (<8.53)	1.0	1.0	1.0
Tertile 2 (≥ 8.53– <9.03)	1.48 (1.20, 1.83) ^**^	1.42 (1.15, 1.76) ^*^	1.31 (1.02, 1.68) ^*^
Tertile 3 (≥ 9.03)	2.56 (2.09, 3.12) ^**^	2.50 (2.05, 3.07) ^**^	1.88 (1.44, 2.44) ^**^
*P* for trend	<0.001	<0.001	<0.001
LnUACR			
Per 1 unit increase	0.29 (0.23, 0.36) ^**^	0.29 (0.22, 0.36) ^**^	0.13 (0.05, 0.21) ^*^
Tertiles of TyG index			
Tertile 1 (<8.53)	0	0	0
Tertile 2 (≥ 8.53– <9.03)	0.12 (0.01, 0.22) ^*^	0.12 (0.02, 0.22) ^*^	0.02 (-0.09, 0.13)
Tertile 3 (≥ 9.03)	0.39 (0.29, 0.49) ^**^	0.39 (0.28, 0.49) ^**^	0.15 (0.03, 0.27) ^*^
*P* for trend	<0.001	<0.001	0.015
UACR ≥ 30 mg/g			
Per 1 unit increase	1.80 (1.51, 2.16) ^**^	1.79 (1.49, 2.15) ^**^	1.43 (1.14, 1.79) ^*^
Tertiles of TyG index			
Tertile 1 (<8.53)	1.0	1.0	1.0
Tertile 2 (≥ 8.53– <9.03)	1.19 (0.85, 1.66)	1.18 (0.85, 1.65)	1.07 (0.75, 1.52)
Tertile 3 (≥ 9.03)	2.06 (1.52, 2.79) ^**^	2.03 (1.50, 2.75) ^**^	1.40 (0.97, 2.02)
*P* for trend	<0.001	<0.001	0.053

Model 1: adjusted for age, sex.

Model 2: No-hypoglycemic and lipid-lowering drugs group adjusted for age, sex, smoke and drink habit, BMI, SBP, DBP, HDL-C, LDL-C, eGFR, self-reported CHD and stroke, antihypertensive drugs; Age <65 years group adjusted for age, sex, smoke and drink habit, BMI, SBP, DBP, HDL-C, LDL-C, eGFR, self-reported CHD and stroke, antihypertensive drugs, hypoglycemic drugs, lipid-lowering drugs.

* p < 0.05;

** p < 0.001.

Other abbreviations as in Tables 1, 2.

## Discussion

In our study, we found the TyG index was significantly associated with arterial stiffness and kidney microcirculation abnormalities. Consistent conclusions were obtained in populations without using hypoglycemic and lipid-lowering drugs, and those aged younger than 65 years old.

The TyG index has been widely recognized as a simple and effective marker for IR, which is associated with the subclinical vascular disease that cannot be explained by conventional cardiovascular risk factors (8, 26–29). Generally, IR perturbs insulin signaling at the level of the endothelial cells, vascular smooth muscle cells, and macrophages, leading to a varying degree of defective vasodilation, oxidative responses, impaired endothelial function, and inflammatory state (30–32), which is associated with increased arterial stiffness. Vascular injury related to IR will develop progressively in asymptomatic subjects during a period of time early from childhood. A long phase of IR and latent vascular injury was proven to precede the clinical onset of T2D and increase cardiovascular risk before the diagnosis of the disease (33, 34). The previous studies showed that IR measured by homeostasis model assessment-IR (HOMA-IR) was associated with aortic stiffness among older adults without diabetes and the middle-aged population (10, 35).

Similarly, the TyG index was independently associated with arterial stiffness measured by baPWV in a relatively healthy Korean population (36), in Chinese hypertensive patients (37), and in Greek postmenopausal women (38). The same conclusion was obtained for baPWV in a cross-sectional study of community-based older adults (39). Moreover, compared with the HOMA-IR, the TyG index was independently and more strongly associated with arterial stiffness in patients with T2DM (40). In addition, a cohort study (13) showed that each one-unit increase in the TyG index was associated with a 39 cm/s increment in the baseline baPWV, and a 29%/year increment of baPWV. In our study, the TyG index was also associated with artery stiffness measured by both cfPWV and baPWV after fully adjusted for traditional cardiovascular risk factors in a general community-based population. So, subjects with a higher TyG index should be aware of the probability of arterial stiffness.

Diabetic nephropathy (DN) is the leading cause of chronic kidney disease in patients initializing renal replacement therapy, and is associated with increased cardiovascular mortality (41). Microalbuminuria was regarded as a marker of micro-circulatory abnormality (42). Several studies suggested that TyG index plays a role in nephric microvascular damage. Srinivasan S et al. (43) showed a higher TyG index was associated with the presence of retinopathy and nephropathy in individuals with diabetes and could be used for monitoring metabolic status in clinical settings. Liu et al. (44) showed the TyG index was independently associated with DN in patients with type 2 diabetes, and was a better marker than HOMA2-IR for the identification of DN in type 2 diabetes patients in a cross-sectional study. In a prospective cohort study (45), the TyG index was significantly higher in patients who developed CKD during the follow-up than in those without CKD (*p* < 0.05). In our study, we found that a higher TyG index was also associated with a higher risk of microalbuminuria.

Few studies have reported associations between both macro- and microvascular damage and the TyG index at the same time. Zhao et al. (39) reported that an elevated TyG index was significantly associated with a higher risk of arterial stiffness and nephric microvascular damage in community-dwelling elderly individuals aged 65 or older in Shanghai, China. In sensitivity analysis, our study further showed consistent results among people aged younger than 65 years old compared to those older ones. Besides, taking into account the influence of medication, we further performed analyses in the population without taking hypoglycemic and lipid-lowering drugs, and reached unanimous conclusions.

## Limitations

There are several limitations of this study. First, this study was a cross-sectional design and it limits the detection of causality between TyG index and long-term clinical outcomes. Second, our findings may not be generalizable to other ethnic groups. Third, Laboratory data such as FPG and TG, were measured only once, may not reflect the true level of the participants. Finally, we did not assess HOMA-IR, which is the gold standard method to measure IR. However, the TyG index is certainly more convenient to be measured in routine clinical practice.

## Conclusion

Triglyceride glucose index was positively associated with artery stiffness measured by both cfPWV and baPWV and nephric microvascular damage measured by UACR, suggesting TyG index may serve as a simple and effective indicator to reflect vascular damage. Further prospective studies are warranted to investigate the associations between the TyG index and the development and progression of TOD.

## Data availability statement

The raw data supporting the conclusions of this article will be made available by the authors, without undue reservation.

## Ethics statement

This study was approved by the Ethics Committee of Peking University First Hospital and it conforms to the provisions of the Declaration of Helsinki. All participants signed written informed consent.

## Author contributions

WJ, YZ, FF, and XW contributed to conception and design of the study and performed partial statistical analysis. WJ wrote the first draft of the manuscript. LG and PS contributed to data collection. JJ organized the database. XW and JL wrote sections of the manuscript. All authors contributed to manuscript revision, read, and approved the submitted version.

## Funding

This study was completed by the Department of Cardiology, Peking University First Hospital and Echocardiography Core Lab, Institute of Cardiovascular Disease at Peking University First Hospital. This project was supported by grant from: (1) National Key Research and Development Program of China, 2021YFC2500500, 2021YFC2500503, (2) Projects of National Natural Science Foundation of China (grant 81703288, 82170452), (3) Capital's Funds for Health Improvement and Research, 2020-2-2053, (4) UMHS-PUHSC Joint Institute for Translational and Clinical Research and the Fundamental Research Funds for the Central Universities, grant No: BMU20160530, (5) Chinese Cardiovascular Association-Access fund, 2019-CCA-ACCESS-112, and (6) Key Laboratory of Molecular Cardiovascular Sciences (Peking University), Ministry of Education and NHC Key Laboratory of Cardiovascular Molecular Biology and Regulatory Peptides.

## Conflict of interest

The authors declare that the research was conducted in the absence of any commercial or financial relationships that could be construed as potential conflicts of interest.

## Publisher's note

All claims expressed in this article are solely those of the authors and do not necessarily represent those of their affiliated organizations, or those of the publisher, the editors and the reviewers. Any product that may be evaluated in this article, or claim that may be made by its manufacturer, is not guaranteed or endorsed by the publisher.

## References

[1] Mancusi C Izzo R di Gioia G Losi MA Barbato E Morisco C. Insulin Resistance the Hinge Between Hypertension and Type 2 Diabetes. High Blood Press Cardiovasc Prev. (2020) 27:515–26. 10.1007/s40292-020-00408-8 32964344 PMC7661395

[2] Hanley AJ Williams K Stern MP Haffner SM. Homeostasis model assessment of insulin resistance in relation to the incidence of cardiovascular disease: the San Antonio Heart Study. Diabetes Care. (2002) 25:1177–84. 10.2337/diacare.25.7.1177 12087016

[3] Khan SH Khan AN Chaudhry N Anwar R Fazal N Tariq M. Comparison of various steady state surrogate insulin resistance indices in diagnosing metabolic syndrome. Diabetol Metab Syndr. (2019) 11:44. 10.1186/s13098-019-0439-5 31223343 PMC6570930

[4] Sánchez-García A Rodríguez-Gutiérrez R Mancillas-Adame L González-Nava V Díaz González-Colmenero A Solis RC. Diagnostic accuracy of the triglyceride and glucose index for insulin resistance: a systematic review. Int J Endocrinol. (2020) 2020:4678526. 10.1155/2020/4678526 32256572 PMC7085845

[5] Singh B Saxena A. Surrogate markers of insulin resistance: a review. World J. Diabetes. (2010) 1:36–47. 10.4239/wjd.v1.i2.36 21537426 PMC3083884

[6] Williams B Mancia G Spiering W Agabiti Rosei E Azizi M Burnier M. ESC/ESH Guidelines for the management of arterial hypertension: The Task Force for the management of arterial hypertension of the European Society of Cardiology and the European Society of Hypertension. J Hypertension. (2018). 36. 10.1097/HJH.0000000000001940 30379783

[7] Ormazabal V Nair S Elfeky O Aguayo C Salomon C Zuñiga FA. Association between insulin resistance and the development of cardiovascular disease. Cardiovascular Diabetol. (2018) 17:122. 10.1186/s12933-018-0762-4 30170598 PMC6119242

[8] Adeva-Andany MM Ameneiros-Rodríguez E Fernández-Fernández C Domínguez-Montero A Funcasta-Calderón R. Insulin resistance is associated with subclinical vascular disease in humans. World J. Diabetes. (2019) 10:63–77. 10.4239/wjd.v10.i2.63 30788044 PMC6379732

[9] van Popele NM Westendorp IC Bots ML Reneman RS Hoeks AP Hofman A. Variables of the insulin resistance syndrome are associated with reduced arterial distensibility in healthy non-diabetic middle-aged women. Diabetologia. (2000) 43:665–72. 10.1007/s001250051356 10855542

[10] Ho C-T Lin C-C Hsu H-S Liu C-S Davidson LE Li T-C. Arterial stiffness is strongly associated with insulin resistance in chinese - a population-based study (Taichung Community Health Study, TCHS). J Atheroscler Thromb. (2011) 18:122–30. 10.5551/jat.5686 21048381

[11] Jia G Aroor AR DeMarco VG Martinez-Lemus LA Meininger GA Sowers JR. Vascular stiffness in insulin resistance and obesity. Front Physiol. (2015) 6:231–231. 10.3389/fphys.2015.00231 26321962 PMC4536384

[12] Markus MRP Rospleszcz S Ittermann T Baumeister SE Schipf S Siewert-Markus U. Glucose and insulin levels are associated with arterial stiffness and concentric remodeling of the heart. Cardiovasc Diabetol. (2019) 18:145–145. 10.1186/s12933-019-0948-4 31684945 PMC6829934

[13] Wu S Xu L Wu M Chen S Wang Y Tian Y. Association between triglyceride-glucose index and risk of arterial stiffness: a cohort study. Cardiovasc Diabetol. (2021) 20:146–146. 10.1186/s12933-021-01342-2 34271940 PMC8285795

[14] Reardon CA Lingaraju A Schoenfelt KQ Zhou G Cui C Jacobs-El H. Obesity and insulin resistance promote atherosclerosis through an IFNγ-regulated macrophage protein network. Cell Rep. (2018) 23:3021–30. 10.1016/j.celrep.2018.05.010 29874587 PMC6082182

[15] Asghari G Dehghan P Mirmiran P Yuzbashian E Mahdavi M Tohidi M. Insulin metabolism markers are predictors of subclinical atherosclerosis among overweight and obese children and adolescents. BMC Pediatr. (2018) 18:368–368. 10.1186/s12887-018-1347-9 30470212 PMC6260656

[16] Manrique C Lastra G Sowers JR. New insights into insulin action and resistance in the vasculature. Ann N Y Acad Sci. (2014) 1311:138–50. 10.1111/nyas.12395 24650277 PMC3989838

[17] Horita S Nakamura M Suzuki M Satoh N Suzuki A Seki G. Selective insulin resistance in the kidney. Biomed Res Int. (2016) 2016:5825170–5825170. 10.1155/2016/5825170 27247938 PMC4876201

[18] Lv L Zhou Y Chen X Gong L Wu J Luo W. Relationship between the TyG index and diabetic kidney disease in patients with type-2 diabetes mellitus. Diabetes Metab Syndr Obes. (2021) 14:3299–306. 10.2147/DMSO.S318255 34305401 PMC8296712

[19] Guo W Zhu W Wu J Li X Lu J Qin P. Triglyceride glucose index is associated with arterial stiffness and 10-year cardiovascular disease risk in a Chinese population. Front Cardiovasc Med. (2021). 8. 10.3389/fcvm.2021.585776 33816569 PMC8017152

[20] Fan F Qi L Jia J Xu X Liu Y Yang Y. Noninvasive central systolic blood pressure is more strongly related to kidney function decline than peripheral systolic blood pressure in a chinese community-based population. Hypertension (Dallas, Tex.: 1979). (2016) 67:1166–72. 10.1161/HYPERTENSIONAHA.115.07019 27141056

[21] S LA A SL H SC Lucy ZY F CA I FH. A new equation to estimate glomerular filtration rate. Ann Intern Med. (2009) 150:604–12. 10.7326/0003-4819-150-9-200905050-00006 19414839 PMC2763564

[22] Zheng M Xu X Wang X Huo Y Xu X Qin X. Age, arterial stiffness, and components of blood pressure in Chinese adults. Medicine. (2014) 93:e262–e262. 10.1097/MD.0000000000000262 25546666 PMC4602627

[23] Zhao F Yang R Maimaitiaili R Tang J Zhao S Xiong J. Cardiac, macro-, and micro-circulatory abnormalities in association with individual metabolic syndrome component: the northern shanghai study. Front Cardiovasc Med. (2021) 8:690521. 10.3389/fcvm.2021.690521 34307503 PMC8298861

[24] Van Bortel LM Laurent S Boutouyrie P Chowienczyk P Cruickshank JK De Backer T. Expert consensus document on the measurement of aortic stiffness in daily practice using carotid-femoral pulse wave velocity. J Hypertension. (2012) 30:445–8. 10.1097/HJH.0b013e32834fa8b0 22278144

[25] Munakata M. Brachial-ankle pulse wave velocity in the measurement of arterial stiffness: recent evidence and clinical applications. Curr Hypertens Rev. (2014) 10:49–57. 10.2174/157340211001141111160957 25392144

[26] Urbina EM Khoury PR McCoy CE Dolan LM Daniels SR Kimball TR. Triglyceride to HDL-C ratio and increased arterial stiffness in children, adolescents, and young adults. Pediatrics. (2013) 131:E1082–E1090. 10.1542/peds.2012-1726 23460684 PMC3608484

[27] Ryder JR Dengel DR Jacobs DR. Jr. Sinaiko AR Kelly AS Steinberger J. Relations among Adiposity and Insulin Resistance with Flow-Mediated Dilation, Carotid Intima-Media Thickness, and Arterial Stiffness in Children. J Pediatrics. (2016) 168:205–11. 10.1016/j.jpeds.2015.08.034 26427963 PMC4698081

[28] Reilly MP Wolfe ML Rhodes T Girman C Mehta N Rader DJ. Measures of insulin resistance add incremental value to the clinical diagnosis of metabolic syndrome in association with coronary atherosclerosis. Circulation. (2004) 110:803–9. 10.1161/01.CIR.0000138740.84883.9C 15289378

[29] Adeva-Andany MM Funcasta-Calderon R Fernandez-Fernandez C Ameneiros-Rodriguez E Dominguez-Montero A. Subclinical vascular disease in patients with diabetes is associated with insulin resistance. Diabetes Metab Syndr. (2019) 13:2198–206. 10.1016/j.dsx.2019.05.025 31235157

[30] Laakso M Kuusisto J. Insulin resistance and hyperglycaemia in cardiovascular disease development. Nat Rev Endocrinol. (2014) 10:293–302. 10.1038/nrendo.2014.29 24663222

[31] Bornfeldt KE Tabas I. Insulin resistance, hyperglycemia, and atherosclerosis. Cell Metab. (2011) 14:575–85. 10.1016/j.cmet.2011.07.015 22055501 PMC3217209

[32] Donato AJ Machin DR Lesniewski LA. Mechanisms of dysfunction in the aging vasculature and role in age-related disease. Circulat Res. (2018) 123:825–48. 10.1161/CIRCRESAHA.118.312563 30355078 PMC6207260

[33] Stehouwer CDA Henry RMA Ferreira I. Arterial stiffness in diabetes and the metabolic syndrome: a pathway to cardiovascular disease. Diabetologia. (2008) 51:527–39. 10.1007/s00125-007-0918-3 18239908

[34] Salomaa V Riley W Kark JD Nardo C Folsom AR. Non-insulin-dependent diabetes-mellitus and fasting glucose and insulin concentrations are associated with arterial stiffness indexes - the aric study. Circulation. (1995) 91:1432–43. 10.1161/01.CIR.91.5.1432 7867184

[35] Poon AK Meyer ML Tanaka H Selvin E Pankow J Zeng D. Association of insulin resistance, from mid-life to late-life, with aortic stiffness in late-life: the Atherosclerosis Risk in Communities Study. Cardiovascular Diabetol. (2020) 19:11. 10.1186/s12933-020-0986-y 31992297 PMC6986071

[36] Won KB Park GM Lee SE Cho IJ Kim HC Lee BK. Relationship of insulin resistance estimated by triglyceride glucose index to arterial stiffness. Lipids Health Dis. (2018) 17:268. 10.1186/s12944-018-0914-2 30474549 PMC6260653

[37] Li M Zhan A Huang X Hu L Zhou W Wang T. Positive association between triglyceride glucose index and arterial stiffness in hypertensive patients: the China H-type Hypertension Registry Study. Cardiovascular Diabetol. (2020) 19:139. 10.1186/s12933-020-01124-2 32948181 PMC7501677

[38] Lambrinoudaki I Kazani MV Armeni E Georgiopoulos G Tampakis K Rizos D. The TyG index as a marker of subclinical atherosclerosis and arterial stiffness in lean and overweight postmenopausal women. Heart Lung Circulat. (2018) 27:716–24. 10.1016/j.hlc.2017.05.142 28690023

[39] Zhao S Yu S Chi C Fan X Tang J Ji H. Association between macro- and microvascular damage and the triglyceride glucose index in community-dwelling elderly individuals: the Northern Shanghai Study. Cardiovasc Diabetol. (2019) 18:95–95. 10.1186/s12933-019-0898-x 31345238 PMC6657056

[40] Wang S Shi J Peng Y Fang Q Mu Q Gu W. Stronger association of triglyceride glucose index than the HOMA-IR with arterial stiffness in patients with type 2 diabetes: a real-world single-centre study. Cardiovasc Diabetol. (2021) 20:82. 10.1186/s12933-021-01274-x 33888131 PMC8063289

[41] Yu D Cai Y Chen Y Chen T Qin R Zhao Z. Development and validation of risk prediction models for cardiovascular mortality in Chinese people initialising peritoneal dialysis: a cohort study. Sci Rep. (2018) 8:1966. 10.1038/s41598-018-20160-3 29386542 PMC5792639

[42] Gowda S Desai PB Kulkarni SS Hull VV Math AA Vernekar SN. Markers of renal function tests. N Am J Med Sci. (2010) 2:170–3. 22624135 PMC3354405

[43] Srinivasan S Singh P Kulothungan V Sharma T Raman R. Relationship between triglyceride glucose index, retinopathy and nephropathy in Type 2 diabetes. Diabetes Metab. (2021) 4:e00151. 10.1002/edm2.151 33532603 PMC7831221

[44] Liu L Xia R Song X Zhang B He W Zhou X. Association between the triglyceride-glucose index and diabetic nephropathy in patients with type 2 diabetes: A cross-sectional study. J Diabetes Investig. (2021) 12:557–65. 10.1111/jdi.13371 33319507 PMC8015837

[45] Xu X Tang X Che H Guan C Zhao N Fu S. Triglyceride-glucose product is an independent risk factor for predicting chronic kidney disease in middle-aged and elderly population: a prospective cohort study. Nan Fang Yi Ke Da Xue Xue Bao. (2021) 41:1600–8. 10.12122/j.issn.1673-4254.2021.11.02 34916184 PMC8685706

